# Supplementary material for: Are carbon emissions trading and green financial instruments synergistic? -Comprehensive quantitative research based on content analysis
Source: PLoS One. 2024 Mar 7;19(3):e0298601. doi: 10.1371/journal.pone.0298601 (PMC10919601; doi:10.1371/journal.pone.0298601)
Supplement: S1 Data — (ZIP) [file pone.0298601.s001.zip › Data/地级市Z_F工作报告.zip!/╔╜╢1⁄2/═■║ú╩╨/└┤╫╘╣1⁄2╓┌║┼í╛┬φ┐╦╩2╛▌═°í┐_2016.docx]

各位代表：

　　现在，我代表威海市人民政府向大会报告工作，请予审议，并请市政协各位委员提出意见。

一、“十二五”时期政府工作回顾

　　过去五年，在市委的坚强领导下，市政府紧紧依靠全市人民，全面贯彻落实党中央、国务院和省委、省政府的决策部署，坚持生态立市、科教兴市、产业强市，主动适应经济发展新常态，妥善应对各种风险挑战，以改革创新的精神扎实做好各项工作，现代化幸福威海建设迈出坚实步伐。

　　——2015年全市生产总值3001.6亿元，“十二五”期间年均增长9.8%，人均超过1.6万美元；地方一般公共预算收入249.7亿元，年均增长14.3%；三次产业比重由7.9：55.9：36.2调整为7.2：47.4：45.4；高新技术产业产值比重预计达到38.5%，提高5.9个百分点，在经济下行压力加大的情况下，实现经济稳健增长、结构持续优化、发展质量与效益同步提高。

　　——万元生产总值能耗下降17%以上，主要污染物减排任务全面完成，城市环境综合整治定量考核稳居全省前列，环境空气质量各项指标保持全省首位，妥善处理经济发展与环境保护的关系，全面提升生态文明建设水平。

　　——常住人口城镇化率由58.2%提高到62.3%；城镇和农村居民人均可支配收入分别超过3.6万元和1.6万元，年均增长10.8%和12.3%；城镇登记失业率保持在1.53%以下；民生支出占财政支出比重由79.5%提高到83.1%，基本公共服务的供给水平更高、覆盖面更广，社会保障水平不断提升。

　　——科学发展综合考核位居全省前列，群众满意度调查评价保持全省第一，城市精细化、智能化管理水平不断提升，群众性精神文明创建取得新突破，以地级市第一名的成绩成功创建全国文明城市。

　　五年来，我们积极应对复杂严峻经济形势，统筹处理稳增长、促改革、调结构、惠民生、防风险的关系，主要做了以下工作。

　　（一）坚持以提高发展质量和效益为中心，打好稳增长调结构主动仗。强化产业强市、工业带动，按照“一产抓特色促跨越、二三产抓结构上规模”的思路，推动产业转型升级。

　　实施产业集群“3+4”发展战略。规划建设医疗器械产业园，获批首个国家碳纤维及复合材料产业基地，新信息、新医药、新材料三大战略性新兴产业集群加速膨胀。支持企业技术改造和品牌建设，机械制造、食品加工、运输设备、纺织服装四大传统优势产业集群加速转型。造船、轮胎、玻璃等行业产能利用率不断提高。“两化”融合、军民结合产业实现突破发展。

　　鼓励新型业态培植和商业模式创新。新建电子商务园区21个，电子商务交易额年均增长39.4%，环翠区成功创建国家电子商务示范基地。新增A级旅游景区30个，成功创建全国旅游标准化示范城市。互联网经济、现代物流、文化创意、休闲产业蓬勃发展，现代服务业占服务业增加值比重提高到41.8%。

　　加快推进蓝色经济区建设。实施渔船更新改造，新增专业远洋渔船230艘，远洋渔业产值年均增长58%，被确定为中国远洋水产品加工与物流基地。建成国家海产品质量监督检验中心、海洋商品国际交易中心，获批中国海参交易中心，被评为中国海洋食品名城。新增国家级休闲渔业基地10个，被评为中国休闲渔业之都、中国钓具之都。入选全国海洋高技术产业基地试点城市。

　　大力发展高效生态农业。推动一产向二产三产跨越延伸，建成国家现代农业示范区。新建现代果园20万亩，特种毛皮产量去年达到1320万张，粮食和主要农产品产量保持稳定，“三品一标”产品产地认定面积比重由35%提高到56%。

　　实施创新驱动发展战略。财政科技投入增长2.2倍，全社会研发经费年均增长15.2%。科技企业孵化器达到42家，孵化面积106万平方米，分别增长9.5倍和13.6倍。支持企业创建省级以上研发平台106家，新建山东船舶技术研究院等15个重大产业创新平台。建成科技创新综合服务平台，促成技术交易3485项。

　　实施英才计划。引进各类人才12万人。新建职业教育示范实训基地49处，培养高技能人才4.4万人，入选首批全国技能振兴百家城市。引进北京交通大学，建成威海海洋职业学院，与110家高校科研院所建立合作关系，促成产学研合作项目405项。发明专利申请量、授权量年均增长17.1%和38.3%。

　　助推大众创业、万众创新。率先创办创业大学，建成32处创业孵化基地；发放创业担保贷款8.4亿元，扶持8649人自主创业，成为首批全国创业先进城市。组织实施“千帆计划”，科技型企业达到1031家，增长5.9倍。实施工商注册制度便利化改革，市场主体总量增长81.5%，其中企业增长78%。

　　（二）抢抓先行先试机遇，全面深化改革开放。主动承担国家、省级改革试点36项，引领带动各领域工作实现新突破。

　　深化投融资体制改革。组建国有资本运营公司，设立天使投资引导基金、产业共赢基金等6支基金，基金总规模221亿元。发行14支城投债和小微企业增信集合债，募集资金138.8亿元。引进各类金融机构30家，扩大科技支行覆盖范围，社会融资总量年均增长38.9%。组建政府性担保公司，设立小微企业贷款风险补偿金，完善续贷过桥周转机制，有效防范企业资金链、担保链风险。实行全口径预算管理。改革重组国有企业27家。新增上市企业、新三板和其他场外市场挂牌企业75家。

　　加强社会领域改革。出台户籍管理、住房保障等改革措施，加快推动农业转移人口市民化，入选国家新型城镇化综合改革试点城市。全面深化农村改革，新增农业新型经营主体3822家。推进基础教育综合改革，义务教育优质均衡发展、校车运营管理、学校管理标准化等国家改革试点实现突破。全面实施县级公立医院改革，被确定为公立医院改革国家联系试点城市。

　　全方位扩大开放。外贸进出口总额去年达到169.4亿美元，一般贸易出口比重由33.9%提高到58.7%，36家企业入选省重点培育国际知名品牌。累计利用外资45.8亿美元，年均增长15.1%。我市企业境外投资14.5亿美元，增长5.3倍。与比利时根特市等4个城市缔结友好城市关系，入选中欧城镇化合作示范城市。

　　全面深化对韩合作。威海与仁川自由经济区被确定为中韩自贸区地方经济合作示范区，获得先行先试重大机遇。实施关检合作“三个一”口岸通关模式，开展电子口岸试点，新增国家一类开放作业港区7个，获批开放肉类、冰鲜水产品等8个特色进口口岸，中韩海运跨境电子商务、中韩海运邮路等政策落地实施。

　　（三）统筹重点区域开发建设，促进市域一体化发展。坚持全域城市化、市域一体化，实施四个“三年突破”战略。

　　协调推进区域发展。文登撤市设区，进入同城化发展新阶段。南海新区成为山东半岛蓝色经济区重点海洋经济新区。东部滨海新城开发建设扎实推进。乳山产业结构调整步伐加快。双岛湾科技城、好运角旅游度假区、乳山滨海新区等重点区域开发建设取得阶段性成果。临港区升级为国家级经济技术开发区，高区、经区加快推进自主创新示范区和产城融合示范区建设。荣成海洋生物产业园成为省级高新区，北海旅游度假区获批省级旅游度假区。荣成被列为全省城乡统筹工作试点市。

　　着力突破交通瓶颈。青荣城际铁路正式通车，去年完成客运量456万人次，地方铁路去年完成客运量244.2万人次。威海机场完成升级改造，旅客吞吐量由82.5万人次提高到132.1万人次。威海港国际客运中心、刘公岛客运中心投入使用，港口货物吞吐量由4866万吨提高到7324万吨。石烟线外移工程、荣文高速公路全线开通。市区两大综合交通枢纽工程投入使用。

　　加快完善城乡基础设施。完成宝泉广场、环翠楼、江家寨立交桥等城建重点工程，新建改造城市道路251条。胶东调水工程建成并正式通水，形成本地水与客水“双保障”的供水格局。新建污水处理厂8座，新铺设污水管网850公里。66.7%的镇实现集中供热，87.5%的镇铺设天然气管道，“户保洁、村收集、镇运输、市处理”的生活垃圾一体化处理体系实现全覆盖。

　　（四）牢固树立绿色发展理念，强化资源环境保护。率先通过省级生态市验收，文登、荣成、乳山被评为国家生态市。

　　实施大气污染防治行动计划。推进散流物体运输、建筑工地扬尘规范化管理，拆除燃煤锅炉141台，燃煤机组全部配建脱硫脱硝除尘设施，加油站全部安装油气回收装置，全面完成黄标车淘汰任务。省测“蓝天白云”天数年均328天，保持全省最多。

　　实施最严格的水资源管理制度。推进市区河道、重点流域、饮用水水源地和地下水污染综合整治。健全海岸带综合管理、沙滩保护等管理制度，实施海域海岛海岸带生态修复工程，完成威海湾海域养殖设施清理，被评为国家海洋生态文明建设示范区。

　　加强生态红线管控。开展闲置低效利用土地清理专项行动，盘活土地2.7万亩。加强山体、林地资源保护，森林覆盖率提高到42.2%。推进里口山城市山地公园建设，新增湿地公园4处。

　　（五）着力提高公共服务能力和水平，持续推进民生改善。扎实办好民生实事，集中力量解决了一批群众关心的实际问题。

　　推动社会保险制度并轨整合。率先实施城乡统一的居民基本养老保险制度、居民基本医疗保险制度，大病保险和大额救助制度并行实施，居民医保最高支付限额提高到50万元。率先实现生育保险城乡全覆盖和生育费用“零负担”。社会保险率先全部实现市级统筹，各项社保待遇保持全省领先水平。

　　完善扶贫济困工作机制。实施精准扶贫项目，减少农村贫困人口3.5万人。建立临时救助制度，救助困难家庭3.5万户次。城乡低保标准分别提高39%和112%。新建公共租赁房、经济适用房1.8万套。建立计划生育特殊困难家庭救助制度。加强残疾人社会保障和服务体系建设，各类助残项目惠及12.1万人次。

　　全面提升教育发展质量。新建改扩建125所公办幼儿园，补助扶持48所普惠性民办幼儿园。加强学校体卫艺教育，培育特色学校85所。实施办学条件标准化提升工程，为147所中小学校配建塑胶跑道和草坪运动场，城乡中小学校全部配齐远程录播教室和交互式多媒体教室，农村中小学新模式校车实现全覆盖。市中小学生综合实践教育中心建成投入使用，被评为全国示范性综合实践基地。实现全国义务教育发展基本均衡县“满堂红”。

　　大力发展公共交通。推动公交回归公益，投资6.7亿元淘汰老旧公交车、建设公交停保场、发展智能公交系统，购置节能环保型公交车880辆。开通大站快线和城际公交快线，各区市全部实施公交客运集约化改造，率先实现城乡公交一体化全覆盖。

　　优化医疗卫生服务。6家医院晋升三级甲等医院，539个村卫生室完成标准化改造。全面推行药品、医用耗材、检验试剂集中配送改革，基层医疗机构全部实施基本药物制度。实施农村妇女和新生儿特定病种免费筛查等一批基本公共卫生服务项目。

　　改善城乡居住环境。开展城乡环境综合整治行动，完成2121个村、107个老旧生活小区整治改造，城乡环卫一体化实现高质量全覆盖。改造棚户区8.2万户，新建农房13.3万户，改造农村危房3.7万户。实施既有建筑供热计量与节能改造4149万平方米。新建城市社区日间照料中心373个、农村幸福院376所，各类养老机构床位达到2.5万张，增长92%。

　　推进智慧城市建设。建成云计算中心和电子政务、工业设计、公共服务三个云平台，实施智能交通、智慧旅游、智慧社保等一批示范项目，成为首批国家智慧城市试点、信息惠民国家试点城市、宽带中国示范城市。开通市民网，启动市民卡工程。推动数字化城管、安全生产网格化监管信息平台、肉菜流通追溯体系建设，运用信息化手段保障公共安全和城市管理服务。

　　加强精神文明建设。大力弘扬社会主义核心价值观，推进美德威海建设，被评为全省“四德”工程建设示范市。实施文化惠民工程，被评为全省首批公共文化服务体系示范区和首批标准化试点城市。承办十艺节“群星奖”戏剧类比赛。被评为全国文化体制改革先进地区和文化强省建设先进市。

　　深入开展全民健身活动。建成市乒羽健身中心，新建城市健身慢行道93.3公里，各区市建设7处全民健身中心，推动学校体育设施向社会开放，城乡社区全部配备体育健身设施，形成15分钟公共健身圈。成功举办长距离铁人三项世界锦标赛和世界杯系列赛，组织仁川亚运会火炬传递活动。

　　深化海陆一体平安建设。健全网格化社会治理体系，加强公共安全视频监控系统建设，社会治安群众满意度位居全省第一。完善道路交通、森林防火、消防管理、应急避难等安全基础设施，加大应急救援装备配备力度，安全事故防范和应急处置能力进一步提高。积极创建国家食品安全城市和农产品质量安全市，“双城联创”模式被国家推广。

　　国防动员、人民防空、民兵预备役工作进一步加强。民族、宗教、妇女儿童、外事、侨务、对台、档案、史志、地震、气象、仲裁、慈善等事业取得新进步。

　　（六）坚持依法行政，大力建设法治政府、服务型政府、廉洁政府。扎实开展党的群众路线教育实践活动、“三严三实”专题教育，持续开展机关作风建设年活动，切实加强政府自身建设。

　　大力推进简政放权、放管结合。实施新一轮政府机构改革和事业单位改革，全面公布行政审批事项目录清单、行政权力清单、政府责任清单，在建设工程审批、市场主体设立等领域推行模块化审批、联审联批模式，取消、下放行政审批事项391项，市级审批事项能精简下放的全部精简下放。

　　依法规范政务运行。组建政府法律顾问团，重大行政决策和规范性文件制订都公开征求意见。加强政府信息公开，扩大预决算公开范围。推进政府信息资源共享和数据公开。开展行政违法预防工作。健全行政复议工作机制。建立行政约谈制度。加强普法依法治理，被评为全国法治城市创建先进单位。

　　主动接受人大的法律监督、政协的民主监督。办理人大代表建议214件、政协提案970件。整合公共服务热线，建立政务服务、应急联动、便民服务三个热线平台。组建群众监督员、网络监督员队伍。各级政府和部门开通政务微博。健全岗位廉政风险防控和防止利益冲突机制，政府系统廉政建设进一步加强。

　　各位代表！刚刚过去的一年，我们坚持稳中求进工作总基调，统筹做好各项工作，全市经济运行总体平稳。全市生产总值增长8.5%，地方一般公共预算收入增长13.1%，城镇和农村居民人均可支配收入分别增长8.6%和8.8%，固定资产投资增长14.1%，社会消费品零售总额增长11%，外贸进出口增长2.2%，其中出口增长11.1%，各项社会事业都取得新进步，实现“十二五”胜利收官。“十二五”规划28项主要指标中，12项约束性指标全部完成，13项预期性指标超额完成，3项预期性指标接近目标值。

　　各位代表！过去五年，是全市上下凝心聚力、攻坚克难、改革创新的五年，也是改革开放步伐迈得更快、转型升级力度更大、人民群众得到实惠更多的五年。政府工作取得的每一点成绩，都倾注了全市上下的心血和汗水，凝聚着社会各界的智慧和力量。在此，我代表市政府，向全市各族人民，向人大代表和政协委员，向各民主党派、工商联和无党派人士、各人民团体，向驻威人民解放军指战员、武警官兵，向各位老领导、老同志，向关心支持威海发展的各界人士和海内外朋友，致以崇高敬意和衷心感谢！

　　各位代表！过去五年，我们始终坚持问题导向，按照逆向切入的思路，下大力气解决经济社会发展中的矛盾和问题。经济发展新常态下，要激发我们这座城市的创新创造活力，仍需要正视问题，在化解矛盾、补齐短板上多下功夫。在增长速度换挡期、结构调整阵痛期，我市企业的抗风险能力、项目建设的支撑力还不够强，新兴产业、新型业态发育不足，新旧动力转换接续不够顺畅；人才、技术、资金等创新资源的集聚能力有待增强，创新驱动的引擎作用尚未得到充分发挥；各类规划的有机衔接与刚性约束有待加强，土地节约集约利用水平有待提高；基本公共服务均等化水平还不够高，新型城镇化面临瓶颈制约等实际难题；部分政府工作人员依法办事、高效服务的意识和能力有待提高，廉政建设、作风建设还需要加强，在一些领域还存在不正之风和违纪违法问题。我们一定强化忧患意识、责任意识，不断加强和改进工作，更加有效地应对各种风险和挑战。

二、“十三五”时期的总体要求和目标任务

　　面向未来五年，我们编制了《威海市国民经济和社会发展第十三个五年规划纲要（草案）》。《纲要（草案）》及《说明》已印发大会，请一并予以审议。

　　“十三五”时期是全面建成小康社会决胜阶段，政府工作的总体要求是：高举中国特色社会主义伟大旗帜，全面贯彻党的十八大和十八届三中、四中、五中全会精神，以邓小平理论、“三个代表”重要思想、科学发展观为指导，深入贯彻习近平总书记系列重要讲话和视察山东重要讲话、重要批示精神，坚持“四个全面”战略布局要求，坚持发展是第一要务，坚持争当全省走在前列排头兵的目标，以创新、协调、绿色、开放、共享发展理念为引领，以提高发展质量和效益为中心，加快形成引领经济发展新常态的体制机制和发展方式，深入实施生态立市、科教兴市、产业强市和全域城市化、市域一体化战略，统筹推进经济建设、政治建设、文化建设、社会建设、生态文明建设，提前全面建成小康社会，实现现代化幸福威海建设新跨越。

　　（一）坚持创新发展，创建国家创新型城市。深入实施创新驱动发展战略，推动经济保持中高速增长、产业迈向中高端水平。在提高发展平衡性、包容性、可持续性的基础上，生产总值年均增长8.5%左右，提前实现比2010年翻一番的目标。正确处理政府和市场的关系，加大结构性改革力度，加快构建有利于创新发展的体制机制，使市场在资源配置中起决定性作用和更好发挥政府作用。实施产业集群“3+4+3”发展战略，培育壮大三大战略性新兴产业集群，改造提升四大传统优势产业集群，加快培育智能装备、海洋生物、物联网三大未来产业。推动生产性服务业向专业化和价值链高端延伸，生活性服务业向精细和高品质转变。构建现代农业产业体系、生产体系、经营体系，促进一二三产业融合发展。推进蓝色经济区建设，深度拓展蓝色经济空间。大力发展军民融合产业，创建国家军民融合创新示范区。以智慧城市建设带动智慧产业发展，促进以互联网为基础的创新创业。坚持走以企业为主体的产学研合作道路，不断提高技术转移转化能力。大力发展科技企业孵化器、众创空间等新型创新创业服务平台，全面提升高端创新要素聚集和承载能力。高区深度融入山东半岛自主创新示范区建设，海洋高新技术产业园实现特色发展。

　　（二）坚持协调发展，打造城市化发展新模式。深入开展国家新型城镇化综合试点，提升中心城区首位度，促进全域城市化、市域一体化发展。坚持以人的城市化为核心，加快推进“三个市民化”，户籍人口城镇化率达到68%左右。坚持生态、生产、生活三生共融，统筹空间、规模、产业三大结构，以主体功能区规划为基础推进“多规合一”，促进产城融合发展。按照适度超前、优化布局、完善网络的原则，有序推动高速铁路、高速公路、城市轨道交通、枢纽场站等重大工程项目，统筹布局地下综合管廊、停车场等市政公用设施，加快建设海绵城市，规划建设一批商贸服务、文化休闲、体育健身等社区便民服务设施，全面提升基础设施现代化水平，让人民群众生活得更方便、更舒心、更美好。

　　（三）坚持绿色发展，打响宜居城市品牌。实施最严格的环境保护制度，全面提升生态宜居水平。实行能源和水资源消耗、建设用地等总量和强度双控行动，严格将各类开发活动限定在资源环境承载能力之内。实施全民节能、节水行动计划，提升发展方式和生活方式绿色、低碳水平。开展海湾、沙滩、海岸线修复整治行动，筑牢生态安全屏障。深入实施大气、水、土壤污染防治行动计划，燃煤热电企业全部实行超低排放，环境空气质量在全省率先全面达到国家二级标准，饮用水水源地水质达标率100%，主要河流水环境全部达到功能区标准，森林覆盖率达到45%左右，共建共享天蓝、地绿、水净、景美的美丽家园。

　　（四）坚持开放发展，打造开放型经济新优势。主动融入“一带一路”等国家开放战略，积极开拓新兴市场，实施精准招商，支持企业“走出去”，推进高水平双向开放。中韩自贸区地方经济合作示范区形成聚集效应，通关便利化、贸易便利化水平全面提高，中韩最便捷贸易通道加快形成，中韩商品交易集散地、中韩合作产业园建设取得突破，在贸易、投资、服务、产业合作等领域形成更多可复制、可推广经验。经区、临港区等开发区加快转型升级，打造联动发展的产城融合示范区、体制机制创新区。

　　（五）坚持共享发展，全面建成小康社会。坚持人人参与、人人尽力、人人享有，不断提高公共服务共建能力和共享水平。市域一体的社会保障体系更加健全，优质均衡的现代化教育发展格局加速形成，覆盖城乡的公共文化服务体系不断完善，率先建成具有示范效应的健康城市。社会治理法治化水平不断提高，公共安全保障体系更加完善。社会主义核心价值观更加深入人心，社会文明程度全面提升。人民生活水平和质量普遍提高，现行标准下农村贫困人口全部脱贫，居民人均可支配收入年均增长8.5%以上，提前实现比2010年翻一番的目标。

　　各位代表！在经济发展新常态下，实现“十三五”规划蓝图，需要我们以百倍的信心应对风险挑战，以时不我待的精神抢抓战略机遇。我们将以脚踏实地的作风、奋勇争先的干劲，全力打好改革创新攻坚战，打赢转型发展的主动仗，以实实在在的工作成效，让人民群众过上更加美好的生活，让威海这座开放城市焕发新的生机、展现新的魅力。

三、2016年政府工作安排

　　各位代表！2016年是实施“十三五”规划的开局之年，是推进结构性改革的攻坚之年，主要预期目标为：全市生产总值增长8%—8.5%，地方一般公共预算收入增长10%左右，居民消费价格涨幅控制在2%左右，城镇登记失业率控制在2%以内，进出口保持稳定，城镇和农村居民人均可支配收入分别增长8.5%左右和9.5%左右，完成省下达的节能减排约束性指标。主要工作任务是：

　　（一）加大结构性改革力度。以产业转型升级为方向，攻克结构性改革难题，切实增强持续增长动力。

　　全面落实五大任务。抓住供给侧结构性改革的关键点，加快去产能、去库存、去杠杆、降成本、补短板。运用市场机制、经济手段、法治办法，引导企业并购重组，加快“僵尸企业”退出。加快淘汰落后产能，化解造船等行业过剩产能。推动化工企业进区入园。引导企业改善房地产供给结构，积极消化房地产库存。组建房源统筹平台，采取租补分离、货币化安置等方式，打通商品房与保障房转换通道。发展住房租赁市场，培育专业化住房租赁企业。积极争取和落实政策措施，加大减费清费力度，降低实体经济企业成本。完善政银企风险处置合作机制，打击非法集资，防范区域性、系统性金融风险。

　　加快制造业改造升级。发挥龙头企业示范带动作用，加快推进医疗器械产业园、碳纤维及复合材料产业基地建设，壮大一批行业“配套专家”企业，大力培育战略性新兴产业。实施工业强基工程，扩大工业技改贴息政策适用范围，推动传统优势产业集群改造升级。对接“中国制造2025”规划，大力发展智能装备产业，推进“机器换人”示范工程。实施103项重点工业投资项目，引导企业持续增加产业领域投资。全社会固定资产投资增长13%左右，先进制造业投资比重进一步提高。

　　突破发展现代服务业。坚持“敲开核桃、一业一策”，推进服务业发展提速、比重提高、水平提升。做优做强生产性服务业，加快发展研发设计、金融保险、文化创意、商务服务、信息技术服务等重点行业。做精做活生活性服务业，积极培育特色商圈，提升体育健身、教育培训、家政服务等产业发展水平，引导形成新的消费需求。社会消费品零售总额增长11%左右。

　　大力发展休闲产业。培育海洋休闲经济，规划建设帆船、游艇基地，打造20处综合性休闲渔业基地和20处海上田园观光体验园区。发展山地休闲旅游，规划建设房车营地。壮大都市休闲农业，新建10处精品观光采摘园。推动文化体验与商业、休闲相融合，打造城市特色文化街区。提升好运角旅游度假区、里口山城市山地公园、昆嵛山国家级自然保护区的保护和开发水平。

　　调整优化农业结构。推广应用农业新技术、新模式，提高水利化、机械化、科技化水平。因地制宜调整种植业结构，增加农产品有效供给。大力发展生态养殖和远洋渔业，建设“海上粮仓”。培育深加工企业和交易市场，促进特色农畜产品种养业稳定发展。开展化肥农药使用量零增长行动。鼓励多种形式适度规模经营，培育新型农业经营主体和服务主体，新增家庭农场120个。

　　大力实施质量和品牌战略。开展质量管理提升行动，引导企业建立首席质量官制度，完善质量管理体系。健全标准创新激励机制，支持企业主导或参与制定行业国家和国际标准。开展知名品牌培育创建工作，支持企业提升研发设计、品牌创建、营销网络拓展能力。发挥名牌企业聚集示范效应，创建优质产品生产基地，提升渔具、地毯等一批特色产品的品牌影响力。

　　实施“互联网+”行动计划。发挥“互联网+”对经济提质增效的促进作用，大力发展“互联网+”协同制造、涉农服务、电子商务、高效物流等产业，培育信息消费、互联网金融、物联网、云计算等新业态。支持企业开展个性化定制、网络化协同制造和服务型制造。搭建胶东半岛城市物流配送云服务平台，构建智能仓储及物流配送体系。争创全省互联网私募股权融资试点。加强信息基础设施建设，推动公共数据资源开放开发。

　　各位代表！企业是经济发展的主体，是推进结构性改革的基础，为企业营造良好发展环境是政府义不容辞的责任。我们将坚持以服务企业发展为己任，尊重企业家、关心企业家、保护企业家，主动问计问需于企业家，多为企业着想，多站在企业的角度思考问题，在制度上、政策上营造宽松的市场经营和投资环境，消除束缚企业手脚的体制机制障碍，让企业在转型过程中轻装上阵，与企业一道攻坚克难，共度难关，共同推动结构性改革，共同提升威海的发展活力和水平。

　　（二）激发创新创业活力。大力营造有利于大众创业、万众创新的发展环境，进一步活跃市场主体，培育新的增长点。

　　大力培植创新型企业。强化企业创新主体地位，实施高新技术企业培育成长计划，新增科技型中小企业500家。扩大科技“创新券”使用范围，对大型仪器共享、科技成果交易、创新咨询等实施补贴。推动发展科技保险，开展科技小额贷款、科技融资担保、专利权融资等创新业务，为创新型企业发展提供融资支持。实施知识产权强基行动，支持企业提高有效发明专利拥有量。

　　支持科技企业孵化器建设。实施科技企业孵化器聚集提升工程，与国内专业孵化机构建立紧密合作关系，吸引高层次孵化人才、创新资金、创新机构，引进专业管理团队加强孵化器专业化运营，提升在孵企业素质与孵化产出。加快推进海洋生物、电子信息等专业孵化器建设。

　　强化创新服务平台建设。加强公共创新平台建设，推动共性技术研发与企业实际需求对接。加快工信部电子信息技术综合研究中心、医疗器械和药品检验检测中心等公共检测平台建设。推动建设碳纤维国家检测中心、医学工程实验中心。支持企业创建省级以上研发平台13个。创建军民科技融合产业协同创新中心。试行科技成果挂牌交易，技术交易额超过27亿元。

　　加强大众创业服务。引导企业和社会机构兴办众创空间，建立威海众创联盟，推动众创、众包、众扶、众筹，聚集各类创业孵化资源。优化创业培训实训机制，实施新一轮大学生创业引领计划，开展创业大赛、创客秀等创业展示活动，创建省级示范创业大学，完成创业实训5000人以上。落实创业补贴和创业担保贷款政策，建设创业服务云平台，满足创业者个性化需求。

　　加快集聚创新型人才。坚持以企业为主体，以重点人才工程为引领，深入实施英才计划，集聚一批对产业转型升级具有带动作用的产业领军人才、科技创业人才、市场营销人才和高技能人才队伍。推进留学人员创业园建设，吸引高端人才来威海创业发展。实施企业家培育计划，培养优秀企业家群体。提高职业教育人才培养质量，培养区域产业发展急需的高技能人才，新建3处示范实训基地。加强与驻威高校共建，深入推进人才引进交流和产学研合作。引进各类人才2万人，培养高技能人才5000人。

　　（三）增创开放型经济新优势。发挥中韩自贸区地方经济合作示范区的聚集效应，推动全方位多元化对外开放。

　　促进对外贸易优进优出。加快自主品牌出口和境外自主营销网络建设，提升出口商品质量、档次和创新要素比重。支持外贸综合服务平台建设，为中小微进出口企业提供便捷服务。实施市场多元化战略，积极拓展非洲、南亚、中东欧等新兴市场。加快发展服务外包，扩大服务贸易进出口。

　　大力实施精准招商。在招商方向上精准定位，推进主导产业补链式招商、新兴产业龙头式招商、传统产业提升型招商，重点引进新型业态和高端项目，引进龙头型、基地型、技术创新型企业，推动本土企业引进战略合作伙伴，提高利用内外资质量效益。在招商方式上精准发力，发挥专业招商队伍作用，制定重点产业招商路线图，开展“量身定做”式合作。实际到账外资增长10%左右，实际利用内资增长15%左右。

　　突破对韩经贸合作。建设电子口岸国际贸易“单一窗口”，扩大中韩优秀进出口企业互认范围。做好离境退税政策实施准备工作。推进海关特殊监管区整合优化改革。加快跨境电商综合服务平台建设。加强与韩国在服务业方面的深度合作。

　　加强全球经济合作。积极参与“一带一路”建设，支持企业安全高效“走出去”。支持企业巩固海外工程承包市场。充分利用外事、侨务、对台资源，扩大经贸文化交流。加强国内区域合作，做好对口支援工作。

　　（四）深化重点领域改革。推动已出台改革方案落实落地，积极谋划新的改革举措，排除影响结构性改革的体制机制障碍。

　　创新投融资机制。推广政府和社会资本合作模式，引导社会资本参与公共基础设施建设和运营。整合市级公共资源交易平台。提高产业投资基金、风险投资基金运营效率，以产业引导、股权投资方式支持产业发展。加强政府债务管理，推进城投债、小微企业增信集合债和专项债券发行，有序做好债券融资工作。

　　引导推动企业改革改制。以管资本为主加强国有资产监管，分类推进国有企业改革，完善现代企业制度。推进供销社综合改革。推动企业规范化公司制改制，对接多层次资本市场，新增上市及新三板挂牌企业10家以上，实现直接融资120亿元。

　　健全财政资金管理机制。完善预算管理体系，建立跨年度预算平衡机制。健全透明预算制度，依法依规公开预决算。全面推进税式支出管理改革。继续清理压减财政专项资金，大力压减三公经费等一般性支出，腾出更多资金用于基本公共服务。

　　强化金融改革创新。完成农村信用社银行化改革。扩大新型农村合作金融试点范围。扩大科技支行覆盖范围、小额贷款保证保险规模，放大小微企业贷款风险资金池效应。提升融资性担保机构的担保和增信能力。拓展保险业服务领域和资金运用渠道。推进大宗商品交易试点，加快国际海洋商品交易中心建设运营。

　　加快推进社会领域改革。深化基础教育综合改革，完善中考招生制度，推进普通高中课程改革，开展非营利性民办学校教师养老保险与公办学校教师同等待遇试点工作。深化城市公立医院综合改革，理顺公立医院管理体制，全面破除以药补医机制。加大政府购买公共服务力度，稳步推动事业单位去行政化。

　　（五）加快推进城市化进程。坚持以人为核心，遵循城市发展规律，着力提高城市发展持续性、宜居性。

　　积极稳妥推进“三个市民化”。实施居住证制度，推动基本公共服务常住人口全覆盖。加快城中村、城边村改造，完成棚户区改造1.9万户，改造旧生活小区28个，新建新型社区21个，推动就地城镇化。推进农村产权交易市场建设，加快农村集体产权制度改革，完善农民土地承包权、宅基地使用权、集体收益分配权有偿转让机制，促进有序流转。

　　大力推动产城融合。深入实施四个“三年突破”战略，推进文登同城化发展和乳山产业结构调整，推动东部滨海新城、临港区、南海新区等重点区域加大产业招商力度，布局一批重点产业项目，强化城市产业发展、吸纳就业和创新能力支撑。

　　推进智慧城市建设。发展公共服务智慧应用，完善城市综合管理信息系统功能，实施智能交通、智慧教育、智慧环保、智慧医疗、智慧文化等示范项目，提高城市管理和服务智能化水平。加快实施市民卡工程，免费发放市民卡50万张。

　　加强基础设施建设。推进文莱高速公路、环山路拓宽改造、蒲石线北段新建等交通项目。实施117项城建重点工程，推动东部滨海新城主干路网及地下综合管廊项目建设。加强公交便民化、绿色化、智能化建设。完成经区污水处理厂扩建、临港区污泥处理二期工程建设。

　　改善农村居住环境。开展美丽乡村行动，完成农村无害化卫生厕所改造9.8万户；改造农村危房1200户；新增规模化供水村326个；改造农村公路150公里。开展农村温暖工程试点，推动燃气、供暖、污水等市政管线向建制镇、新型社区延伸。

　　（六）着力优化生态环境。严格执行生态红线制度，促进资源节约高效利用，进一步提高环境质量。

　　加强水资源保护利用。强化水生态环境治理，推进雨污分流设施建设，加强水功能区和排污企业监管。严格饮用水水源地保护，推进泊于水库新建、八河水库增容等工程建设。完成3个小流域和4条城郊河道综合整治。实施农村河塘综合整治工程，加固病险塘坝242座。加强地下水资源管理。严格水资源消耗总量和强度控制，推广高效节水技术，建设节水型社会。

　　实施空气质量全面优化行动计划。推动工业绿动力计划，加快分散燃煤锅炉治理，完成22台热电企业燃煤锅炉超低排放改造任务，引导企业提高煤炭清洁高效利用水平。抓好企业挥发性有机物治理。强化成品油监管，严格机动车尾气排放检测。加强餐饮业油烟污染整治。推动秸秆综合利用。深入开展工地扬尘、散流物体运输、裸露地面专项治理。

　　强化生态功能区保护。加强森林公园、湿地公园、自然保护区、风景名胜区、海洋保护区等生态功能区建设。加强海洋环境保护国际合作，探索减少垃圾流入海洋的示范机制。提高土地和海域节约集约利用水平。加强农业面源污染防治。深入开展绿化工程，成片造林8万亩。

　　（七）着力优化公共服务。贯彻共享发展理念，强化政府职责，切实增加公共服务供给。集中力量办理26项民生实事，解决群众关心、社会关注的实际问题。

　　实施脱贫攻坚工程。按照精准扶贫、精准脱贫的要求，统筹推进专项扶贫、行业扶贫和社会扶贫，推动扶贫项目进村到户，促进转移就业脱贫。建立特困人员供养制度、重特大疾病医疗救助制度，加强因病致贫、因残致贫家庭救助。

　　开展精准就业促进活动。落实援企稳岗政策，建立失业预防调控机制，加强就业援助，帮助高校毕业生、就业困难人员、农村劳动力等群体稳定就业，新增城镇就业3.3万人，转移农村劳动力1.5万人。构建和谐劳动关系，维护职工和企业合法权益。

　　完善社会保障体系。实施全民参保计划，提高社会保险待遇水平。新建城市社区养老服务中心20处。大力推广医养融合发展模式，发展康复护理型养老机构，新增机构养老床位3000张。实施残疾人奔小康工程，推进十大助残项目，为2.5万名残疾人提供保障和服务。新增公共租赁住房514套。

　　支持教育优先发展。新建、改扩建学校31所，进一步缩小中小学班额。新建、改扩建公办幼儿园12所，支持普惠性民办幼儿园发展。大力发展体卫艺教育，开工建设市青少年活动中心，支持中小学生社团开展活动，推进足球、游泳、帆船运动进校园。

　　推进健康城市建设。支持镇卫生院、社区卫生服务中心建设国医堂。完善康复医疗体系，建立慢性病早期干预服务机制。实施全面二孩政策。开工建设市游泳馆，建设完善各区市全民健身中心。推行医保个人账户支付健康消费办法。举办铁人三项、帆船帆板、山地自行车等赛事，广泛开展全民健身活动。

　　大力加强文化建设。深入开展精神文明创建活动，实施文明市民培育工程，推进美德威海建设，积极践行社会主义核心价值观。实施优秀传统文化教育普及工程。完善基层综合性文化服务中心建设，推进公共文化服务体系标准化建设。加快建设市图书馆、群众艺术馆、档案馆、方志馆。实施广播电视节目无线数字化覆盖工程。支持文化、体育类协会和社团开展群众性文化体育活动。加快影视文化产业园、油画艺术原创基地建设。

　　（八）加强和创新社会治理。深化海陆一体平安建设，推进社会治理精细化，保持社会和谐稳定。

　　提高社会治理法治化水平。扎实开展“七五”普法工作。实施法律服务惠民、法律援助利民、矛盾调处安民“三大工程”，推动法律服务向基层延伸。发挥信访、仲裁作用，健全社会矛盾排查预警和调处化解综合机制。加强城乡社区建设，实现政府治理和社会调节、居民自治良性互动。

　　加快社会信用体系建设。建设公共信用信息平台，整合信用信息资源，统筹推进信用威海建设。建立以守信激励和失信惩戒为重点的社会信用体系运行机制，推动在行政管理事项中率先使用信用信息报告。实施重点领域信用示范工程，争创全省社会信用体系建设试点市。

　　强化公共安全保障。加强公共安全视频监控系统建设，健全立体化社会治安防控体系。推进安全生产网格化监管，健全隐患排查治理和安全预防控制体系，落实企业安全生产主体责任。实施生命安全防护工程，减少道路和车辆事故。扎实推进“双城联创”，开展“守护舌尖安全”整治和药品、中药材专项治理，保障人民群众饮食用药安全。加强防灾减灾能力建设，强化应急教育培训、装备配备和预案演练，提高突发事件应对处置能力。

　　加强民族、宗教、妇女儿童、地震、气象、慈善工作。做好双拥共建和优抚安置工作，加强国防动员、民兵预备役、打击走私和人民防空建设。

四、加强政府自身建设

　　“十三五”期间，继续大力建设法治政府、服务型政府、廉洁政府，坚持依法行政、高效施政、廉洁从政，更好地承担起改革发展责任。

　　（一）更加严格按照法定权限和程序行使权力，依法履行政府职责。各级政府及工作人员带头遵守宪法和法律，不断提高法治意识和依法行政能力。坚持法定职责必须为、法无授权不可为，继续大力推进简政放权、放管结合，健全政府依法决策机制，依法制定政府规章和规范性文件，在法治轨道上推动政府各项工作。健全政府信息公开制度，依法公开重点领域政府信息，让行政权力在阳光下运行。加强行政违法预防和行政执法监督，强化行政复议规范化建设，做到严格规范公正文明执法。强化勇于负责、敢于担当的责任意识，坚决纠正不作为、乱作为，坚决治理懒政、怠政和失职、渎职问题，切实做到公正用权、公平处事。

　　（二）更加主动优化行政服务，为群众创造便捷高效的服务环境。坚持把群众满意作为一切工作的出发点和落脚点，出台加强服务型政府建设意见，进一步创新行政服务方式，让政府服务更加高效、更加规范、更加贴心。推行行政机关首问负责制，实施服务质量公开承诺制，把联动服务、规范服务、亲情服务推广到所有公共服务领域，让群众切实感受到政府部门优化行政服务的努力和变化。持续推进作风问题专项整治，切实解决“门难进、脸难看、话难听、事难办”等问题，严肃整治行政审批和行政服务中的不正之风，让群众不托人也能好办事、快办事、办成事。

　　（三）更加坚决打好反腐倡廉攻坚战，切实加强政府系统廉政建设。全面落实党风廉政建设责任制，强化“一岗双责”，严格执行廉洁自律准则和纪律处分条例，践行“三严三实”要求，推进作风建设常态化、长效化。持续开展廉政警示教育活动，强化防止利益冲突工作，对公共资金、国有资产、国有资源和领导干部履行经济责任情况实行审计全覆盖，严控岗位廉政风险，把权力制约的制度围栏扎得更紧更牢。持之以恒正风肃纪，严肃查处发生在群众身边的“四风”和腐败问题，严肃查办各类违纪违法案件，保持惩治腐败高压态势。

　　各位代表！政府的一切权力来自人民，必须对人民负责，接受人民监督。我们将自觉摆正位置，以人民为尊、以人民为重，坚持为了群众要真心、依靠群众要真诚、服务群众要真干，带着感情开展服务，努力把工作干到人民群众的心坎上；自觉接受监督，以积极主动的姿态，更好地接受人大的法律监督、政协的民主监督、媒体的舆论监督、群众的社会监督，更及时地回应人民群众的关切，努力使各项工作都充分体现人民群众的意愿；自觉履行承诺，以对人民群众高度负责的态度，扎实做好各项工作，努力建设人民满意政府。

　　各位代表！肩负全面建成小康社会的历史重任，我们踏上“十三五”的新征程。让我们更加紧密地团结在以习近平同志为总书记的党中央周围，在市委的坚强领导下，凝心聚力，奋发有为，以改革创新的精神、真抓实干的作风，不断开创各项事业发展新局面，为实现现代化幸福威海建设新跨越而努力奋斗！
